# Repeat Coronary Angiography in Patients Aged over 50 Years with Previously Normal/Non-Obstructive Coronary Angiogram—Insights from a Retrospective Study

**DOI:** 10.3390/jcm13030870

**Published:** 2024-02-02

**Authors:** Ariel Roguin, Ofer Kobo, Simha-Ron Meisel, Ziad Darawsha, Mahmood Odeh, Aharon Frimerman, Naama Amsalem, Rami Abu Fanne

**Affiliations:** 1 Department of Cardiology, Hillel Yaffe Medical Center, Affiliated to the Bruce Rappaport School of Medicine, Technion-Israel Institute of Technology, Hadera 3200003, Israel; arielr@hy.health.gov.il (A.R.); oferk@hymc.gov.il (O.K.); meisel@hy.health.gov.il (S.-R.M.); ziadd@hymc.co.il (Z.D.); aharonf@hymc.gov.il (A.F.); naamaa@hy.health.gov.il (N.A.); 2 Emergency Department, Hillel Yaffe Medical Center, Affiliated to the Bruce Rappaport School of Medicine, Technion-Israel Institute of Technology, Hadera 3200003, Israel; mahmoudo@hymc.gov.il

**Keywords:** normal angiogram, non-significant coronary disease, atherosclerosis, atherothrombosis, myocardial infarction

## Abstract

(1) **Introduction**: A significant proportion of patients undergoing coronary angiography (CAG) have normal (NCA) or non-obstructive coronary artery disease (NOCAD). This study retrospectively tested the incidence of re-catheterization, and long-term outcomes of this population in patients aged over 50 years. (2) **Methods**: We identified all patients above 50 years of age with NOCAD who underwent their first CAG at our center between January 2008 and December 2019. Patients were evaluated for their baseline characteristics, risk factors profile, and indication for CAG. Patients undergoing repeat CAG after the index procedure were assessed for the above, including the primary preventive pharmacotherapy prescribed. (3) **Results**: A total of 1939 patients were reported to have NOCAD. Of these, 1756 (90%) patients (62% males, median age 66 (56–75) years) had no repeat angiography (group 1). Repeat angiography was performed in 10%: 136 (7%) proved futile (median time for repeat angiography 5 (3–8) years) (group 3), and 47 (3%) ended with angioplasty (median time for repeat angiography 4 (3–6) years) (group 2). Male gender, BMI above 30 (23% vs. 13%), hypertension (68% vs. 57%), diabetes (28% vs. 17%) and smoking (36% vs. 19%) were significantly higher in the interventional group. Regression analysis showed both paroxysmal atrial fibrillation and hyperlipidemia were significantly associated with repeat CAG. The indication for the first CAG was mainly symptoms related. In the interventional repeat angiography (*n* = 47) the incidence of troponin positive cases increased from 8.2% before intervention to 57.5%, 50% being ST elevation cases. The symptoms-related cases went from 36.7% to 18.4%. Intriguingly, 85% of the interventional group were not prescribed statin and/or aspirin on a regular basis, and/or did not adhere to treatment. (4) **Conclusions**: NOCAD is a frequent occurrence. The threshold for repeat angiography must be higher, better reserved to troponin positive cases. Moreover, patients must be handled according to their risk profile, not being mistakenly reassured by a snapshot benign coronary angiography.

## 1. Introduction

Coronary atherosclerosis is a major cause of morbidity and mortality worldwide [1]. Atherosclerosis is a complex, multifactorial pathological process that is the result of numerous risk factors including advanced age, hypertension, diabetes, hyperlipidemia, obesity, smoking, and chronic low-grade inflammation. Lipid streaks in arterial walls gradually develop into atheromatous plaques. Disruption of the plaques capsule might culminate in partial or total occlusion of the affected artery. Atherosclerosis is vascular narrowing, systemically involving the heart, peripheral arteries, and the central nervous system, and it usually starts in the teens and 20s [2,3]. Invasive coronary angiography is the gold standard for identifying anatomical epicardial narrowing. Virtually, the yield of this test in contemporary elective patients is low, frequently demonstrating luminally normal or non-significantly narrowed epicardial arteries [4]. In fact, non-obstructive coronary artery disease (defined as <50% stenosis in all vessels) is an increasingly recognized condition, with a prevalence ranging from 50% to 72% in patients presenting with stable angina/nonspecific chest pain, and up to 20–30% among patients sustaining acute coronary syndrome [5,6,7,8,9,10]. Moreover, invasive coronary angiography is not considered a sensitive modality for detecting coronary atherosclerosis. For instance, 48% of the patients labeled as normal in angiography proved atherosclerotic by Intravascular Ultrasound (IVUS) [11].

Progressive coronary narrowing is highly unpredictable, ranging from slowly progressive atherosclerotic lumen loss, to unstable, thrombotic acute occlusion [12]. The most prevalent indication for coronary angiography is a clinical scenario compatible with cardiac angina, with up to 42% of the angiograms performed disclosing NOCAD [13]. This fact emphasizes the limitation of stand-alone clinical judgment during patients’ management.

The ACC/AHA Guidelines have outlined the indications for coronary angiography in specific conditions [14]. Initially, patients with chest pain-induced recurrent hospitalization who have abnormal (yet not high risk) or equivocal findings in noninvasive testing were given a class IIb indication for CAG. The authors stated that the findings of a normal coronary angiogram in such patients indicate a good long-term prognosis that is reassuring to both the patient and the physician. They further mentioned that studies have indicated that a normal angiogram in this setting significantly reduces symptoms and subsequent hospitalizations.

A previously documented NOCAD was integrated in the structured clinical decision-making process under the recommendation for CAG in unstable coronary syndrome, when “recurrent chest discomfort suggestive of unstable angina but without objective signs of ischemia and with a normal coronary angiogram during the past 5 years” was given only class III indication for CAG.

Practically, this population is prone to ongoing healthcare utilization and is frequently sent to repeat, redundant angiographical procedures. Moreover, patients with NOCAD is frequently reassured that the origin of their chest pain is not cardiac and are offered no specific preventive management/treatment beyond reassurance [15,16]. Although traditionally perceived to have favorable prognosis, successive evidence demonstrates a frequent persistence of chest pain, with recurrent hospitalization, and with a not-infrequently documented progression to obstructive coronary artery disease and adverse cardiac events among NOCAD patients relative to age- and sex-matched reference subjects [7,8,16,17]. Jespersen et al. showed elevated risk for major cardiovascular events among patients with chest pain and established NOCAD, compared to reference population free from ischemic heart disease [8]. NOCAD was further proved to be associated with increased risk for myocardial infarction and all-cause mortality [17]. The WISE study group [18] followed patients, mainly women, with NOCAD, and found a high incidence of mortality and repeat angiogram (18% and 19%, respectively, at 10 years). The grim prognosis of this group has caused some to advocate for a change in the philosophy regarding this group by proceeding with further meticulous tests targeting microvascular dysfunction and trying to identify a subgroup at high risk for future cardiovascular events [19]. Contrary to the latter, the SCAAR group demonstrated a low mortality rate of 0.3–0.4% at two years follow-up, among stable angina patients with documented NOCAD [6].

These discrepancies raise questions regarding the real natural history and prognosis of this group.

The current retrospective study aimed to evaluate all patients above 50 years old with their first CAGs performed at the Hillel Yaffe medical Center (HYMC) between 2008–2019 with the diagnosis of NOCAD for prognostic factors including repeat heart catheterization, its indication, and the characteristics of patients with repeat/interventional angiography relative to the rest.

## 2. Methods

### Study Group

In a cohort study we retrospectively evaluated all patients above 50 years old, presented between 1 January 2008 and 31 December 2019, with a first coronary angiogram demonstrating NOCAD. As atherosclerosis is essentially a gradual, lifelong continuum of changes in arterial tissues, we thought a normal/non-obstructive coronary tree at the advanced age of above 50 years might display a lifelong insurance against future progression. NCA was defined as coronary tree with entirely smooth, non-stenotic lumen, and NOCAD was defined as <50% stenosis in any of the epicardial coronary arteries. Patients were excluded if they had previous CAD. The study was conducted in accordance with the Declaration of Helsinki and approved by the HYMC Institutional Review Board (protocol code is 79-10-HYMC) with a waiver of the need for informed consent due to its retrospective nature.

## 3. Data Collection

A dataset of patients’ demographics and baseline characteristics including age, gender, weight, height, and classical CAD risk factors (hypertension, hyperlipidemia, diabetes, renal function, and family history of ischemic heart disease) was retrieved from the medical records. HYMC operates a fully electronic patient medical chart system that includes the clinical, laboratory, and radiological data. It also has access to the Israeli population records; hence, any mortality is automatically updated. The final coronary angiographic diagnosis was classified as NCA or NOCAD. Coronary narrowing was assessed using the quantitative coronary analysis (QCA, SyncVision version 4.1.0.5, Philips Volcano, Zaventem, Belgium). In the case of repeat documented CAG, the interval between the procedures was recorded. The indication for each procedure was recognized (chest pain, aortic/mitral valve related, coronary CT, chest pain and ECG changes/positive troponin/wall motion abnormalities at echocardiogram/positive ergometry/positive scan, or CPR), and the repeat procedures were classified as interventional (balloon angioplasty or stent implantation) or non. The first hospital discharge letter for each patient with repeat interventional CAG was retrospectively checked for the presence of the following items: prescribed medication at the time of discharge, and clear recommendation on smoking cessation. In addition, these patients’ files were evaluated for medication adherence and smoking status at readmission.

## 4. Statistical Analysis

Differences between the three groups according to the patients’ age were tested with ANOVA. To adjust for multiple comparisons for categorical parameters, Pearson’s chi-squared test was used. The ROC model was used to find the best cutoff for predicting redo of catheterization. A logistic regression model was used to predict redo of catheterization with adjustment to gender, smoking, DM, HTN, hyperlipidemia, and paroxysmal atrial fibrillation (PAF); *p* < 0.05 was consider as significant. SPSS version 28 was used for all statistical analysis.

## 5. Results

A total of 1939 eligible patients with a NOCAD angiogram were included: 1756 (90.5%) patients with no repeat angiogram (62% men, median age 66 (56–75) years), 136 (7%) patients with repeat angiogram demonstrating NOCAD (60% men, median age 66.5 (58–73) years), and 47 (2.5%) patients with repeat angiogram which was interventional (83% men, median age 62 (59–72.5) years). Patients’ characteristics in each group are attached in Table 1.

One can appreciate a higher risk profile in the interventional group relative to the non-repeat CAG group, which includes: male predominance (83% vs. 62%, *p* = 0.003), obesity (23% vs. 13%, *p* = 0.067), diabetes (28% vs. 17%, *p* = 0.07), hyperlipidemia (74% vs. 54%, *p* = 0.007), paroxysmal atrial fibrillation (PAF) (4% vs. 1.5%, *p* = 0.16), and smoking (36% vs. 19%, *p* = 0.008). It is noteworthy that 85% of the interventional group were initially labeled as NOCAD, as opposed to 25% of the non-interventional, repeat angiography group. A logistic regression model (Table 2) used to predict the occurrence of repeat catheterization with adjustment to all categorical variables found both Paroxysmal atrial fibrillation and hyperlipidemia to be significantly associated with recurrency, with odds ratios of 3.9 (95% CI 1.8–8.5, *p* < 0.001) and 2.5 (95% CI 1.7–3.6, *p* < 0.001), respectively.

**Figure 1:**
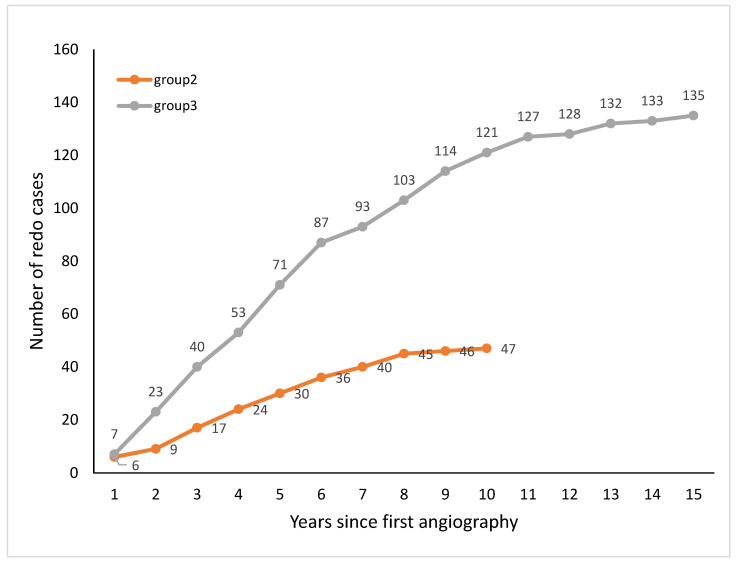
The cumulative annual occurrence of repeat CAG in groups 2 and 3. Group 2—repeat interventional angiography, Group 3—repeat non-interventional angiography.

The cumulative annual occurrence of repeat CAG in groups 2 and 3 is presented in graph 1. The median (IQR) time from first CAG to first repeat CAG was 5 (3–8) and 4 (3–6) years in the NO-PCI and PCI groups, respectively (Figure 1).

**Figure 2:**
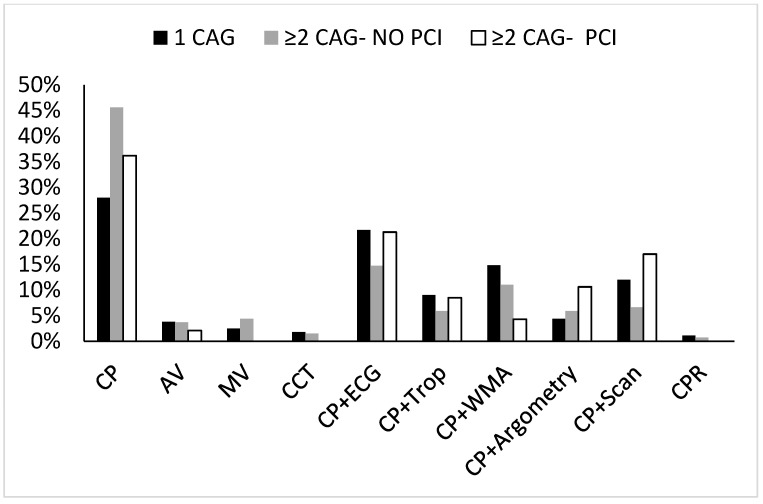
The distribution of each indication for the first angiography in the study groups. CAG—coronary angiography, CP—chest pain, WMA—wall motion abnormalities, AV—aortic valve, MV—mitral valve, Scan—thallium scan, CCT—coronary CT, CPR—cardiopulmonary resuscitation.

In addition, we tested for the indication of the first CAG in each group. Figure 2 illustrates the differences between the groups in relation to the first CAG indication.

Overall, a troponin-negative/symptoms-directed CAG was reported in 81%, 85%, and 89% of the single CAG, and the first angiogram of the ≥2CAG NO-PCI and ≥2CAG PCI groups, respectively, *p* = NS. The proportion of troponin positive cases was 9%, 6%, and 9%, respectively (Figure 2).

We next evaluated the indications for repeat CAG in both the interventional and non-interventional arms.

**Figure 3:**
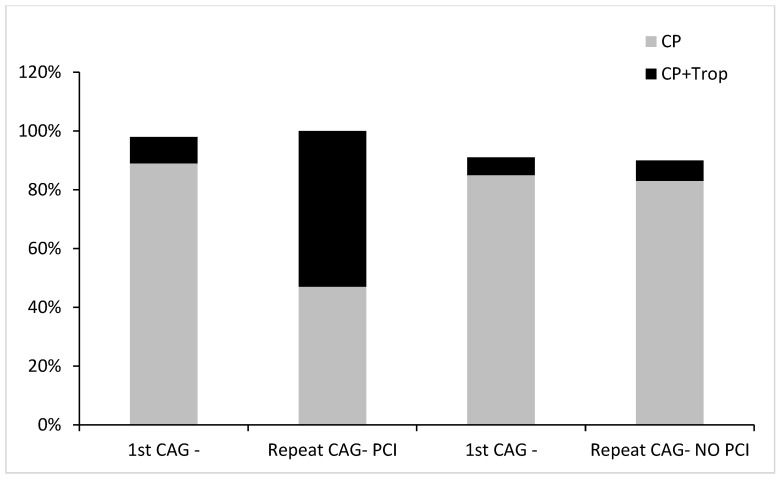
The differences in the indications between the first CAG and the repeat CAG in the repeat non-interventional and interventional subgroups, respectively. CP—chest pain, Trop—troponin.

Figure 3 unequivocally shows that the overall distribution of the indications for CAG did not change in the repeat, non-interventional group (troponin-negative, symptoms-directed CAG in 85% vs. 83%, *p* = 0.33), as opposed to a significant increase in the proportion of troponin-positive cases in the interventional group, from 9% in the first CAG to 53% in the repeat interventional CAG, *p* < 0.001. Remarkably, 50% of the troponin-positive group were STEMI. The proportion of troponin-negative-directed CAG in the interventional subgroup went down from 89% to 47%, *p* = 0.01.

Retrospective assessment of the readmission reports for the regular preventive pharmacotherapy used disclosed that in the interventional repeat CAG group, 100% of the diabetic, 69% of the hypertensive, and 20% of the hyperlipidemic patients were on regular treatment, and only 8.5% were routinely on aspirin therapy. On the other hand, in the diagnostic repeat CAG group, 95% of the diabetic, 83% of the hypertensive, and 70% of the hyperlipidemic patients were on regular treatment, and 70% were routinely on aspirin therapy. We finally tested the 3-year mortality rate in the different groups showing 2.9%, 2.1%, and 1.5% mortality rate in group 1, group 2, and group 3, respectively.

## 6. Discussion

NOCAD is reported in 20–70% of the total coronary angiograms, depending on the primary indication for the procedure [5,6,7,8,9,10]. The natural history and prognosis of patients aged over 50 years with NOCAD is controversial. Particularly, the incidence, timing, and indications for repeat CAG in this group are ill reported.

Conceptually, a major drawback of NOCAD results is that they end with a reassuring message, while disregarding the importance of primary prevention measures.

Effective management of this population requires a clear understanding of two distinct coronary pathophysiological processes. The first is atherosclerotic artery disease, which involves medium and large size arteries and is considered the leading cause of mortality and morbidity in developed countries [20]. Atherosclerosis is a slowly progressing clinical syndrome. The initiation and progression of atherosclerosis is a function of the cumulative exposure to a multitude of recognized, mostly modifiable risk factors. In a naturally evolving atherosclerotic course, it takes about 10 years for new de novo lesions to present, and nearly 5 years for non-significant plaque to manifest [21]. At the other end of the spectrum there is the acute ruptured plaque phenomenon which generally ends with acute myocardial infarction. Previous studies confirmed that 30–50% of acute ST elevation myocardial infarction cases occurred in previously non-significantly stenotic segments [22,23,24]. This finding illuminates that atherosclerosis and atherothrombosis are two distinct, however overlapping, pathophysiological processes. A recent work by Rowe and colleagues [25] identified 6068 patients with a reported first normal angiogram, of whom 162 had a repeat angiogram at follow-up. They excluded 65 patients who were re-analyzed and classified as non-significant CAD. The remaining patients’ median age was 60 y; 40% were males, 85% had diabetes, 66% had hypertension, and 68% had dyslipidemia. During a median follow-up of 51 months, and despite heightened classical risk profile, only 10% showed some degree of atherosclerosis progression; nevertheless, none were considered significant. The authors ended with a reassuring message stating that normal coronary arteries rarely progress to significant disease within 4 years. Nevertheless, multiple constraints limit the reliability of this message; first, most of the patients were women (60%). Second, the vast majority of the group (5904) lacked any follow-up, including cardiovascular mortality. Third, although the risk factors profile was described, no clues considering the control and/or the medical treatment of these risk factors were provided. Finally, the classification of the angiograms as normal, with the exception of 65 patients who were not reported, have a potential for bias. The classification of coronary arteries as normal based on angiograms was proved inaccurate and misleading; a previous study using IVUS modality detected atherosclerosis in nearly 48% of the angiographically labeled normal angiograms [11].

In our study the incidence of repeat CAG was 9.5%; of them 25.7% (2.5%) were interventional, at a median of 4 (3–6) years. Interestingly, 85% of the interventional cases were initially classified as NOCAD, and most (57.5%) were readmitted for troponin positive event (median time (IQR) was 5 (3–7) years), 50% being ST segment elevation MI (median time (IQR) was 5 (3.75–7) years). In other words, 57.5% patients manifested with primary atherothrombotic sequel of CAD, and 46.8% progressed to significant sclerotic narrowing, both at similar time intervals, with the initial labeling of NOCAD in the vast majority of the cases, making the last near-essential prerequisite for a future event, and pointing the remarkable negative predictive value of angiographically NCA labeling for future coronary events. The positive predictive value of positive troponin test at readmission was also previously proved in patients with first event of myocardial infarction (MI) with non-obstructed coronary arteries (MINOCA) [26]. Recurrent MI after MINOCA signifies a subgroup with a more extensive coronary atherosclerosis, often eventually requiring epicardial angioplasty. Among others, our observation stresses the crucial role of atherothrombosis, which may involve a previously luminally non-significant/normal segment [20,21,22]. Our observation is in line with previous ones showing a higher progression of coronary narrowing in patients initially labeled as NOCAD compared to NCA [25,27]; the reported clinical event rate was 1% per year in the NCA group compared to 4.3% in the NOCAD group (*p* < 0.001).

We also found that the interventional subgroup demonstrated a higher risk profile, including a higher occurrence of smoking, diabetes, hyperlipidemia, obesity, family history of IHD, and male predominance. Notwithstanding their heightened risk profile, this group demonstrated inferior pharmaco-preventive coverage. The baseline characteristics of the repeat non-interventional CAG group are very similar to the repeat interventional group (Table 1); nevertheless, their primary prevention pharmacotherapy was remarkably superior, primarily their lipid-lowering drugs (70% vs. 20%) and micropirin prescription (70% vs. 8.5%).

A recent study retrospectively assessed patients who underwent two successive diagnostic angiograms and showed age, hematocrit, cigarette smoking, hypertension, and stable and unstable angina diagnosis to be independent predictors of atherogenesis [28].

Among others, people are generally reluctant to take drugs while asymptomatic, and non-pharmacologic preventive interventions applying lifestyle modifications could potentially be helpful [29]; the latter was out of the scope of our retrospective study design. Other limitations in our study design are worth mentioning: we did not review patients’ angiographic films to verify the precise classification of NCAs vs. NOCAD. The same is applicable to the repeat angiograms. Additionally, the data regarding patients’ pharmacotherapy at the different stages was obtained from their hospitalization medical reports, which might possibly be biased. Moreover, assessing the level of control for each risk factor is out of the scope of the current study. Lastly, we did not compare the interventional group pharmacotherapeutic approach to other groups. These facts shed a light on a possible explanation for the grim prognosis of some patients previously labeled as NOCAD. Atherosclerotic/thrombotic risk is a continuum and cumulative and patients must be managed according to their baseline risk profile in an attempt to halt CAD progression.

## 7. Conclusions

Overall, our study has two major take-home messages: first, the diagnostic label of NOCAD carries an excellent prognosis in most of the patients, particularly patients with NCAs, and the threshold for repeat CAG should be higher than non-labeled individuals, rather than applied when myocardial necrosis/atherothrombosis and/or ST elevation are confirmed at readmission. Second, in addition to the reassuring message for patients with firstly documented NOCAD, we must not underestimate vulnerable patients with already proven risk factors for CAD, particularly those with angiographically documented NOCAD. NOCAD can potentially be inversely associated with cardiovascular risk stratification; however, the pretest probability for cardiovascular events and recurrent myocardial infarction should never be overlocked. Greater attention to the spectrum of CAD risk and application of patient education strategies, including nicotine cessation, lifestyle modification, and risk profile-directed pharmacotherapy, may result in better patient outcomes.

## Figures and Tables

**Table 1:** Demographics and baseline characteristics of the study patients. CAG = coronary angiography, PCI = percutaneous coronary intervention, HTN = hypertension, DM = diabetes mellitus, IHD = ischemic heart disease, NA = not applicable, PAF = paroxysmal atrial fibrillation, group 1 = No repeat CAG, group 2 = repeat CAG-PCI, group 3 = repeat CAG-NO PCI.

	Group 1; *n* = 1756	Group 2; *n* = 47	Group 3; *n* = 136	*p*-Value
Age	65.8 ± 12.9	64.7 ± 11.05	65.9 ± 11.9	*p* = 0.83
Gender Male Female	1089 (62%) 667 (38%)	39 (83%) 8 (17%)	82 (60%) 54 (40%)	*p*^1^ = 0.003 *p*^2^ = 0.71 *p*^3^ = 0.004
Smoking	333 (19%)	17 (36%)	44 (32%)	*p*^1^ = 0.008 *p*^2^ = 0.0005 *p*^3^ = 0.72
DM	298 (17%)	13 (28%)	33 (24%)	*p*^1^ = 0.07 *p*^2^ = 0.046 *p*^3^ = 0.69
HTN	1018 (58%)	32 (68%)	96 (71%)	*p*^1^ = 0.18 *p*^2^ = 0.004 *p*^3^ = 0.85
Hyperlipidemia	948 (54%)	35 (74.5%)	104 (76.5%)	*p*^1^ = 0.007 *p*^2^ < 0.001 *p*^3^ = 0.84
Renal failure	10 (0.6%)	0	0	NA
Obesity	228 (13%)	11 (23%)	22 (16%)	*p* = 0.067
PAF	26 (1.5%)	2 (4%)	8 (6%)	*p*^1^ = 0.16 *p*^2^ = 0.002 *p*^3^ = 1.00
Family IHD	151 (8.6%)	6 (13%)	11 (8%)	*p* = 0.58

*p*^1^—group 1 vs. group 2; *p*^2^—group 1 vs. group 3; *p*^3^—group 2 vs. group 3.

**Table 2:** The occurrence of repeat catheterization with adjustment to all categorical variables.

	B	*p*-Value	Odds Ratio	95% C.I. for EXP(B)	
				Lower	Upper
Gender	0.302	0.074	1.353	0.972	1.883
Diabetes	0.332	0.079	1.394	0.962	2.020
Hypertension	0.154	0.410	1.166	0.809	1.681
Hyperlipidemia	0.916	<0.001	2.500	1.706	3.664
Paroxysmal atrial fibrillation	1.369	<0.001	3.931	1.810	8.536

## Data Availability

All data presented in this manuscript are available from the corresponding author upon reasonable request.
